# Dynamics of ionized poly(4-hydroxystyrene)-type resist polymers with *tert*-butoxycarbonyl-protecting group

**DOI:** 10.1038/s41598-024-67794-0

**Published:** 2024-07-20

**Authors:** Kazumasa Okamoto, Yusa Muroya, Takahiro Kozawa

**Affiliations:** 1 SANKEN (The Institute of Scientific and Industrial Research), Osaka University, Ibaraki, Osaka 567-0047 Japan; 2 Artificial Intelligence Research Center, SANKEN (AIRC-SANKEN), Osaka University, Ibaraki, Osaka 567-0047 Japan

**Keywords:** Chemically amplified resist, Pulse radiolysis, Radical cation, Deprotonation, Density functional theory, Materials chemistry, Physical chemistry, Polymer chemistry, Theoretical chemistry

## Abstract

The imaging reactions of resist materials used for nano-patterning have become radiation-chemical reactions, with the shortening of wavelengths of the exposure light sources in lithography systems. The most widely used patterning materials in industrial lithography are chemically amplified resists (CAR). Understanding the deprotonation mechanism of ionized polymers (radical cations) is important for acid generation in CARs. In this study, the dynamics of radical cations in poly(4-hydroxystyrene) (PHS)–type resist polymers, partially and totally protected by *tert*-butoxycarbonyl (*t*-BOC) groups, are investigated using a combination of electron pulse radiolysis experiments, acid yield measurements, and quantum chemical calculations. The *t*-BOC(oxy) group exhibits π-electron-donating behavior in the monomer cation but changes to electron-accepting behavior in the polymer cation, owing to the interaction between substituents. The destabilization of radical cations due to decreased intramolecular charge resonance may contribute to the high deprotonation efficiency of *t*-BOC-capped PHS polymers.

## Introduction

Extreme ultraviolet (EUV) lithography has been increasingly applied in manufacturing cutting-edge semiconductor products since its introduction into high-volume production lines. It provides high-resolution, low-cost patterning that improves the performance of semiconductor products^1^. Chemically amplified resists (CAR)^2,3^ have been used for decades in conventional ArF (193 nm) and KrF (248 nm) photolithography as well as in EUV lithography (13.5 nm). Next-generation EUV resists with high sensitivity, low defects (low roughness), and high resolution (< 10 nm) can be realized by focusing on the development of CARs or novel resists, along with the processing platform^4–8^. In addition, the high resolution of CAR patterning in electron-beam (EB) lithography is crucial for photomask fabrication^9,10^ and nanopatterning^11,12^. CARs generally consist of a base polymer, a photoacid generator (PAG), and other additives (such as base quenchers for controlling the acid diffusion length). In EUV and EB lithography, the radiation-induced chemical reaction, *i.e.*, the ionization of the base polymer, and the sensitization of secondary electrons to the PAG trigger acid generation^13–15^. The generated acids serve as catalysts to remove the protecting group from the polymer, generally by the application of thermal energy. The deprotection process changes the polarity of the polymer and subsequently its solubility in the developer, creating a dissolution contrast between the exposed and unexposed areas, which leads to creation of patterns. Understanding the molecular-level dynamics associated with resist radiation chemistry is important for achieving sub-10 nm resolution, sub-nanometer roughness, and defect-free patterning. The mechanism of acid generation after resist ionization is as follows^13,14^:1

2 $${\text{PAG}} + {\text{e}}^{ - } \to {\text{products}} + {\text{ X}}^{ - } ,$$

3 $${\text{Polymer}}^{ \cdot + } + {\text{e}}^{ - } \to {\text{Polymer}}^{*} ,$$

4 $${\text{Polymer}}^{ \cdot + } \to {\text{Polymer}}^{ \cdot } + {\text{H}}^{ + } ,\;{\text{and}}$$

5 $${\text{H}}^{ + } + {\text{X}}^{ - } \to {\text{H}}^{ + } {\text{X}}^{ - } .$$

Here, Polymer, Polymer^·+^, Polymer^*^, Polymer^·^, e^−^, PAG, X^−^, and H^+^X^−^ represent the resist polymer, radical cations of the resist polymer, excited states of the polymer, neutral polymer radicals, secondary electrons, PAG molecules, counter anions of the acid, and acid molecules, respectively. Among these reactions, the deprotonation of the polymer radical cation is an important elementary reaction that, along with the reaction of PAG with electrons, increases the quantum yield of acids. Polystyrenes (PS) are aromatic molecules whose radical cations exhibit π-electron reactivity. Radical cations exhibit a larger *p*Ka in polar solvents than their neutral state molecules^16^. In addition, as electron donation to the phenyl ring increases, radical cations become more stable and their deprotonation efficiency decreases. Although radical cations of phenolic monomer compounds^17,18^ and polymers^19,20^ have been shown to induce deprotonation even in nonpolar media, the details of deprotonation from radical cations in resist polymers with bulky protecting groups remain poorly understood^21^.

This study investigated the dynamics of radical cations of poly(4-hydroxystyrene) (PHS) and fully and partially *tert*-butoxycarbonyl (*t*-BOC)-protected PHS as typical base polymers of CARs. In the study, pulse radiolysis was conducted under room-temperature conditions and combined with quantum chemical calculations to reveal the dynamics of radical cations in polymers. Electronic and steric substituent effects of polymeric radical cations were clarified for gaining insights into the design of next-generation resists.

## Experimental and calculations

### Polymer samples

**Figure 1:**
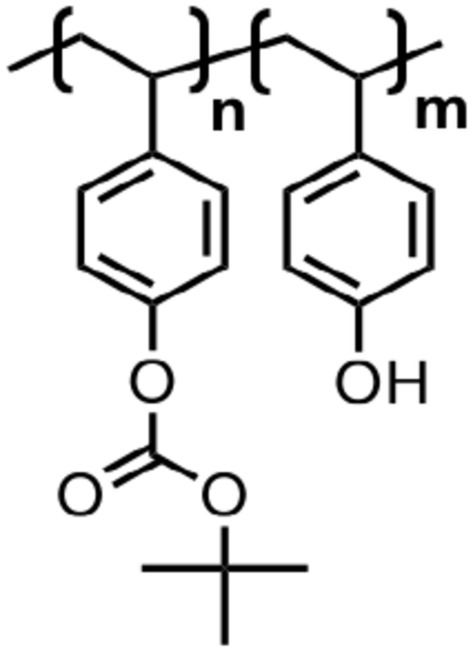
Polymer structures used in this study.

Polymers were purchased from suppliers and no further purification process was performed. The structures of the polymers used in this study are shown in Fig. 1. Used polymers were both homopolymers [poly(4-(*tert*-butoxycarbonyl)oxy)styrene (PTBOS) with Mw = 12,700) and poly(4-hydroxystyrene) (PHS) with Mw = 11,000] and copolymers [partially protected PHS by *t*-BOC [P(TBOS-*co*-HS)], synthesized from PHS (Sigma-Aldrich, Mw = 11,000) by Nard Institute]. The 4-(*tert*-butoxycarbonyl)oxy)styrene (TBOS) monomer composition in the copolymers was 29.1%, 54.6%, and 76.0%.

### Pulse radiolysis measurements

In the pulse radiolysis method, the photoabsorption of short-lived intermediates produced through radiolysis by EB pulse irradiation was measured. The samples were Ar-saturated PHS, P(TBOS-*co*-HS), and PTBOS solutions in cyclohexanone (CHN) and 1,2-dichloroethane (DCE). The optical cell containing the liquid sample was coaxially irradiated inversely with a synchronized EB pulse and analyzing light from a xenon flash lamp. The time resolution of the kinetic traces was approximately 10 ns in the wavelength range of 300–1600 nm. Irradiated EB pulses (26 MeV, 8 ns) were generated by the L-band linear accelerator at SANKEN, Osaka University.

### Acid yield quantification in polymer films

The initial acid yields in EB-irradiated polymer films were quantified by titration using coumarin 6 dye (C6, Sigma-Aldrich). The absorption maximum of C6 shifts from 460 to 533 nm upon addition of protons (acid)^22^. First, sample solutions were prepared. The solution consists of a polymer, PAG [triphenylsulfonium nonaflate (TPS-nf, Midori Kagaku)], and C6 in a mass ratio of 10:1:0.5, dissolved in tetrahydrofuran (THF). The solution was cast onto a quartz wafer and fabricated into a film using the spin-coating method. Absorption spectra were measured in air after exposure to 50 keV EB (100 μC/cm^2^; Hamamatsu, EB-ENGINE) *in vacuo* (< 70 Pa) using an absorption spectrometer (Jasco, V-550). The acid yield was determined by measuring the absorbance of the proton adduct of C6 (λ = 533 nm) normalized by the thickness of the sample film by transmission.

### Density functional theory calculations

Density functional theory (DFT) (ωB97X-D/def2-TZVP level) was used to calculate optimized model conformations of radical cations as a portion of the polymer chain. Ethylbenzene and isotactic (*m*) 2,4-diphenyl pentane derivatives were evaluated as monomer and dimer (diad) models. Ionization energies for the dimer and monomer models were calculated from the difference in formation standard Gibbs energies of radical cations and neutral molecules after structural optimization. In addition, natural bond orbital (NBO) analysis for neutral and radical cation models and time-dependent DFT (TD-DFT) calculations for radical cations were performed. All DFT calculations were performed using the Gaussian16 Rev.C program^23^. The molecular structures were displayed in the Winmostar program^24^.

## Results and discussion

The pulse radiolysis method is a pump–probe method that can measure the dynamics of intermediates produced when a sample is irradiated with high-energy beams (*e.g.*, electron, ion, X-ray). In addition, by examining the optical absorption spectra, events related to transient chemical species and electronic transitions could be identified. By using 26 MeV EB pulses, even centimeter-sized optical cells containing condensed phase samples could be irradiated, delivering nearly uniform energy along their path. The kinetic information of intermediate species could be observed by measuring the intensity of analytical white light synchronized with EB pulses. In this study, kinetic traces after EB irradiation were observed over time with approximately 10 ns time resolution. The experiments were performed on the sample polymer solutions, namely PHS, P(TBOS-*co*-HS), and PTBOS solutions. The primary process of the radiation-induced chemistry in the resist polymer solutions in CHN under Ar saturation is considered to be as follows^19^:6

7 $${\text{c}} - {\text{hex}}^{ \cdot + } + {\text{Polymer}} \to {\text{Polymer}}^{ \cdot + } ,$$

8 $${\text{Polymer}}^{ \cdot + } + {\text{e}}^{ - } \to {\text{Polymer}}^{*} ,$$

9 $${\text{Polymer}}^{ \cdot + } \to {\text{Polymer}}^{ \cdot } + {\text{H}}^{ + } ,$$

where c-hex, c-hex^·+^, and c-hex^*^ represent CHN molecules, radical cations of CHN, and the excited states of CHN, respectively. In the EB-irradiated solution, first, the solvent molecules are mainly ionized (forming solvent radical cations and secondary electrons) and/or electronically excited. Then, positive holes migrate to the polymer with lower ionization energy or redox potential. Although the polymer in the solid film is directly ionized (Eq. 1), the radical cations of the solute polymers in the solution are mainly formed indirectly.

**Figure 2:**
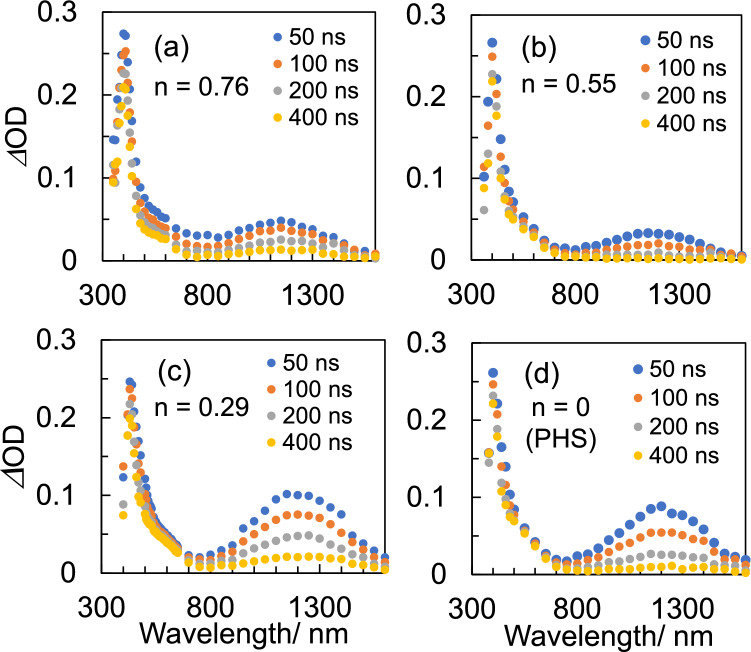
Transient absorption spectra obtained by pulse radiolysis of 500 mM (unit conc.) polymers in Ar-saturated CHN: (**a**, **b**, **c**) P(TBOS-*co*-HS) and (**d**) PHS. Here, *n* is the percentage of OH groups of P(TBOS-*co*-HS) protected by *t*-BOC groups: (**a**) 0.76, (**b**) 0.55, and (**c**) 0.29.

The transient absorption spectra of PHS-based polymers in CHN obtained by pulse radiolysis after 8 ns of EB irradiation are shown in Fig. 2. The absorption maxima around 400 nm can be identified as a phenoxy radical based on the previous results of pulse radiolysis of PHS solutions^19,20,25^. The peak intensities are similar, as shown in Fig. 2. The formation of phenoxy radicals through the deprotonation of the radical cation of phenolic molecules in nonpolar media has two proposed mechanisms. The first is an ultrafast path (< 1 ps) that occurs immediately after formation of the radical cation. The second is a slower path that occurs in the nano- to microsecond range^17,18^. The absorption of phenoxy radicals produced during the 8 ns EB pulse irradiation is induced by ultrafast deprotonation immediately after ionization, which is thought to occur in a similar manner, regardless of the copolymerization ratio of the polymer. This consistency may be attributed to the spread of charge resonance (CR) to the phenyl ring moiety of multiple monomer units in the polymer radical cations formed after ionization, with the vinylphenol unit acting as a proton source^26^. CR formation associated with conformational relaxation in PS radical cations occurs sufficiently fast (< 40 ps^27^) compared to the nanosecond timescale. Deprotonation from the vinylphenol unit was confirmed in the radical cation of poly(4-hydroxystyrene-*co*-styrene) copolymers^26^. Assuming that the styrene moiety, which has the lower deprotonation efficiency in the copolymer, is ionized first, efficient deprotonation from the hydroxyl group is induced by CR when the vinylphenol moiety is within 2.5 units. The broad absorption bands in the near-infrared (NIR) region with absorption maxima at 1200 nm shown in Fig. 2 are identified as CR bands of dimer or multimer radical cations^28,29^, where positive holes are delocalized across multiple aromatic monomer units^19,30–32^. The copolymer with a *t*-BOC protection ratio of *n* = 0.76 displays an additional absorption in the wavelength region of 450–700 nm in Fig. 2a. This absorption is attributed to the short-lived species associated with TBOS units, as it is observed in polymers with a high percentage of TBOS. More details will be discussed later, alongside the results of pulse radiolysis of PTBOS solutions.

**Figure 3:**
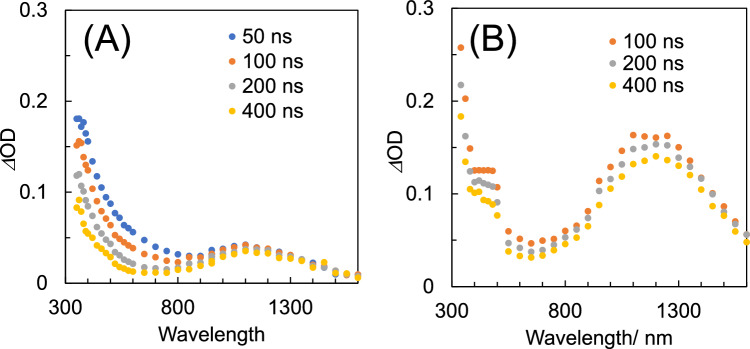
Transient absorption spectra obtained by pulse radiolysis of 100 mM (unit conc.) PTBOS in Ar-saturated (**A**) CHN and (**B**) DCE.

Next, the transient absorption spectra obtained by pulse radiolysis of the PTBOS solution are shown in Fig. 3. In the CHN solvent [Fig. 3A], absorption of phenoxy radicals is not observed. However, significant absorption in the ultraviolet–visible region (A-band) and the CR band in the NIR region is observed. Halocarbon solvents have been widely used in radiation chemistry to produce radical cations in solutes^31,33,34^. PHS has low solubility in halocarbons, whereas PTBOS has both low polarity and high solubility in halocarbons. The transient absorption spectra in the visible region in DCE [Fig. 3B] differ significantly from those in CHN, indicating a clear shoulder at approximately 450 nm. This absorption around 450 nm can be attributed to the local excitation (LE) band of the multimer radical cations^35,36^ or the charge transfer (CT) complex or ion pair formed by the recombination of the PTBOS radical cations with the Cl^−^ produced by the dissociative electron attachment of DCE, as observed in the pulse radiolysis results of PS derivatives^31,33^. In DCE, electrons react immediately with the DCE molecule, and the electrons are replaced by anions with lower mobility. As a result, electron–hole recombination reactions between the solvent and/or polymer radical cations and electrons are suppressed. This suppression leads to a greater yield of radical cations in the time region (> 10 ns), as observed by the nanosecond pulse radiolysis shown in Fig. 3. The decay of PTBOS radical cations in CHN is slower than in polymers containing vinylphenol units, with a half-life longer than 400 ns. This suggests that the deprotonation of PTBOS radical cations is slower than that of the vinylphenol-containing OH group as a result of substitution by the *t*-BOC group. The decrease of the A-band observed in CHN is accelerated by the addition of an electron scavenger (TPS-nf) (Fig. S1), and the lifetime is longer than the emission lifetime from the singlet excited state (~ 10 ns). Therefore, the A-band can be identified as a radical anion of the polymer unit protected by *t*-BOC. Previous pulse radiolysis results of the PTBOS solution in air-saturated CHN using a variable temperature system suggested the A-band is due to cation absorption^21^. However, considering its slight reactivity with cation scavengers, the present results indicate that the A-band is unlikely to be a cation species.

**Figure 4:**
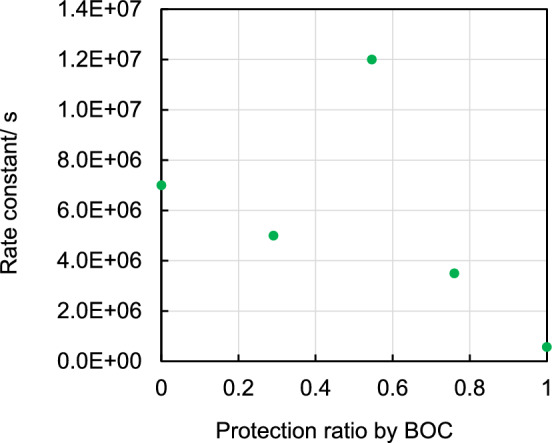
Relationship between the protection ratio (*n*) and decay rate constant of the CR band at 1200 nm.

**Figure 5:**
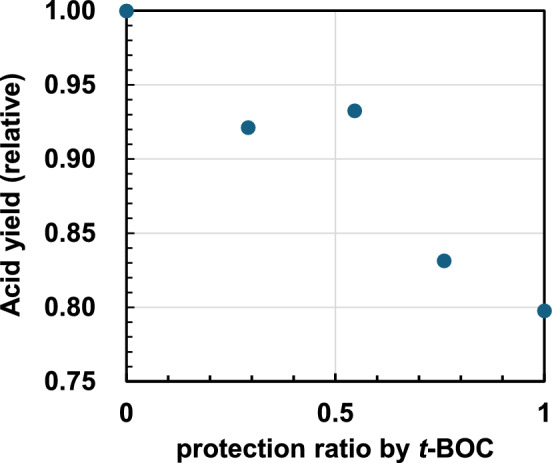
Relationship between the protection ratio (*n*) and relative acid yield in the polymer film obtained by the C6 titration method.

The relationship between the decay rate constant of the CR band absorption at 1200 nm in CHN and the *t*-BOC protection ratio of PHS is also shown in Fig. 4. The decay rate constants tend to decrease with increasing *t*-BOC protection ratio but are specifically enhanced around a 50% protection ratio. Next, the acid (deprotonation) yield produced after 50 kV EB irradiation in the polymer film was also measured, and the results are shown in Fig. 5. The acid yield correlates well with the rate constant for deprotonation of radical cations in pulse radiolysis and tends to decrease with increasing protection ratio, showing a local maximum around a 50% protection ratio. Additionally, the acid yield in the PTBOS film is approximately 80% that in the PHS film. The *t*-BOC protection ratio of the polymer before EB irradiation was confirmed by Fourier-transform infrared spectroscopy (FT-IR) measurement (Fig. S2).

Next, the effects of *t*-BOC and OH group substituents on radical cations of styrene derivatives modeled as monomer units were analyzed by quantum chemical calculations using DFT. As a model for radical cations formed in polymers, 2,4-diphenylstyrene derivatives as a diad (dimer) model were also investigated, including interactions between phenyl rings. For simplicity, isotactic (*m*) tacticity was applied in the dimer model. For the monomer and dimer models, the ionization energies and electron-donating behavior of *p*-substitution by *t*-BOC(oxy) or OH groups were evaluated. The polarity of the PHS thin films is relatively low [relative permittivity (*ε*) = 4.1^37^], which may be further decreased with the addition of the* t*-BOC groups. Therefore, the stabilizing effect of radical cations by solvation was to be small and calculations were performed under vacuum conditions for simplicity. Since the Hamett parameter, which has been widely used as a descriptor of electron-donating behavior, is not applicable to radical cationic molecules, the pEDA descriptor proposed by Dobrowolski et al. was used instead^38^. The values of the pEDA descriptor represent the charge difference between substituted and unsubstituted molecules in a π-valence electron system using the NBO charge determined by quantum chemical calculations. The calculation results are shown in Table 1. In the monomer model, both *t*-BOC(oxy) and OH substituents for the neutral molecule and radical cation exhibit electron-donating behavior (positive pEDA value). Here, pEDA was calculated relative to ethylbenzene (pEDA = 0). In addition, the ionization energy, *i.e.*, the difference between the Gibbs free energy of the radical cation and the neutral molecule, reduces by the presence of electron-donating substituents. The calculated ionization energy of ethylbenzene agrees well with the experimental value for ethylbenzene (8.6–8.8 eV)^39^.

**Table 1:** Ionization energy (IP) and pEDA of the monomer and dimer models, energy difference (Δ*G*^0^) between conformations (anti, syn), distances between phenyl rings, CR band wavelength, and oscillator strength (*f*) of the dimer cation model in PHS, PTBOS, P(TBOS-*co*-HS), and polystyrene (PS) obtained by DFT and TD-DFT calculations.

	PHS (anti)	PHS (syn)	PTBOS (anti)	PTBOS (syn)	P(HS-TBOS)	PS
IP (monomer)/ eV	7.86		7.86		–	8.53
pEDA (neutral monomer)	0.08		0.04		–	0
pEDA (monomer cation)	0.21		0.90		–	0
ΔG^0^(dimer cation)/ eV	0	0.019	0.008	0	–	–
IP(dimer)/ eV	7.25	7.29	7.35	7.29	7.08	7.85
Interplanner distances/ nm	0.325	0.326 (0.36*)	0.327	0.338 (0.36*)	0.344 (0.36*)	0.326
pEDA (neutral dimer)	0.011	0.013	− 0.724	− 0.131	− 0.040	0
pEDA (dimer cation)	0.030	0.024	− 1.284	− 0.256	− 0.148	0
TD-DFT(dimer cation) (λ/nm)	1106.6	1103.1	1097.1, 992.4	1094.3	713.3, 681.9	951.9
TD-DFT(dimer cation) (*f*)	0.130	0.124	0.0827, 0.024	0.095	0.023, 0.008	0.145

*Results of B3LYP/6–31 + G level calclations^21^.

**Figure 6:**
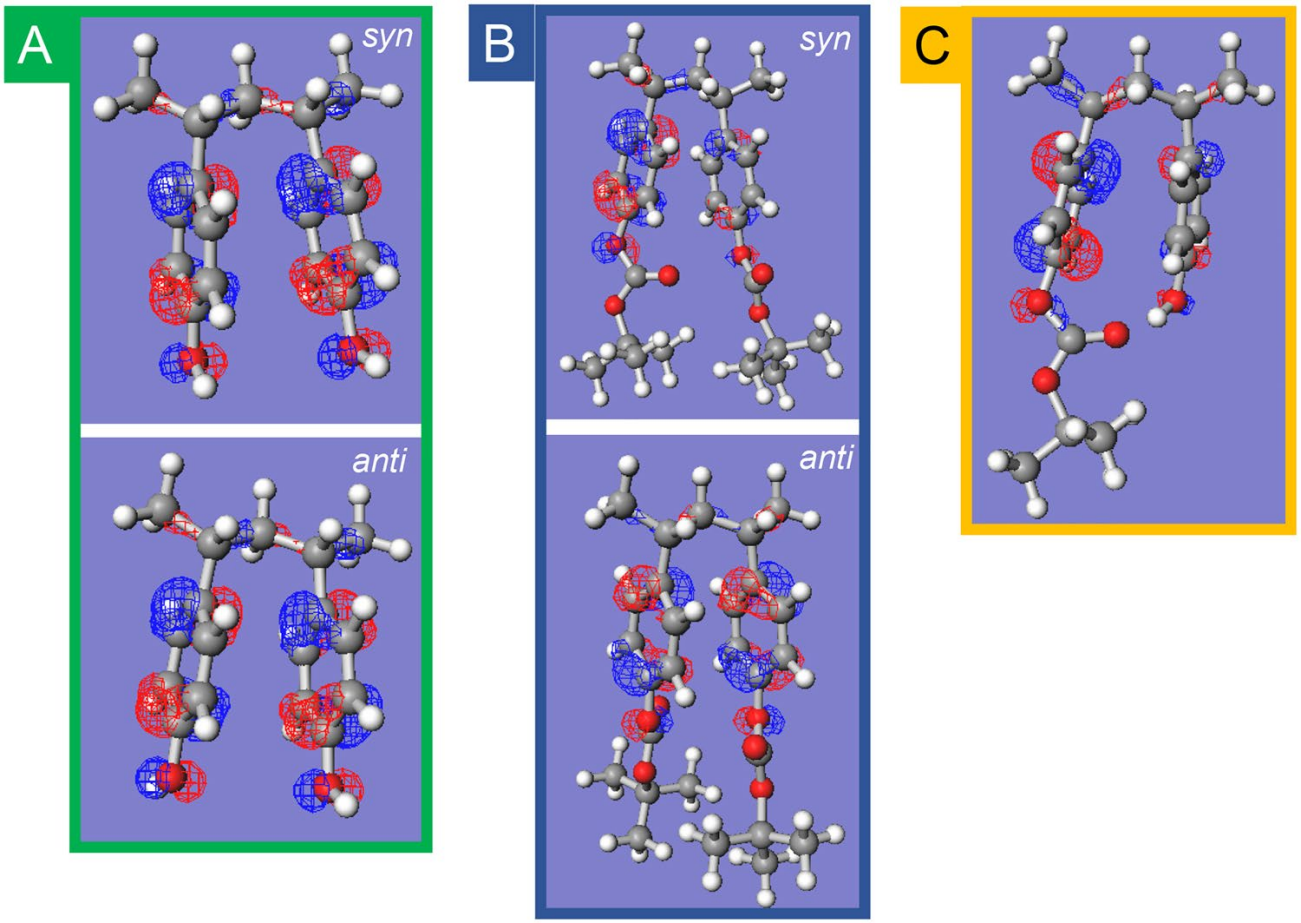
Optimized structures of dimer cation model compounds [2,4-diphenyl pentane derivatives (*m*, *m*)] with SOMO (isovalue = 0.05). The three combinations of substituents of the phenyl ring (X, Xʹ) in the dimeric model are (**A**) (OH, OH), (**B**) (*t*-BOC, *t*-BOC ), and (**C**) (OH, *t*-BOC). In (OH, OH), the direction of the hydroxyl group and, in (*t*-BOC, *t*-BOC), the direction of the carbonyl oxygen are indicated as *syn* for the same direction and *anti* for the opposite direction. All DFT calculations are performed using the ωB97X-D/def2-TZVP level.

The intramolecular dimer radical cation models (*p*-substituted 2,4-diphenylstyrene derivatives) optimized using DFT calculations are shown in Fig. 6. NBO and spin charge maps are also shown in Figs. S3–S7. Three combinations of substituents (X and X′) on the two phenyl rings of the derivatives were analyzed: (X, X′) = (OH, OH), [*t*-BOC(oxy), *t*-BOC(oxy)], and [*t*-BOC(oxy), OH]. For the (OH, OH) model in Fig. 6A, two stable conformations were obtained depending on whether H atoms of the OH group were oriented in the same (*syn*) or opposite (*anti*) direction. Similarly, for the [*t*-BOC(oxy), *t*-BOC(oxy)] model in Fig. 6B, stable conformations were found based on whether the O atoms of the carbonyl group were oriented in the same (*syn*) or opposite (*anti*) direction. The two stable structures were obtained from the initial configuration of the substituent orientation. The energy differences between the respective *syn* and *anti* conformations in the dimer model and the interplanar distances between the benzene rings are presented in Table 1. The pEDA values were determined from the difference in the sum of the charges of the π electrons of the NBO charges on the carbon atoms of the benzene ring in 2,4-diphenylbenzene and its derivatives. These results are also presented in Table 1. The energy differences between the *syn* and *anti* conformations are less than *k*T (0.026 eV at 298 K), indicating a free conformational change between the two. Differences in the distances between benzene rings are negligible for (OH, OH) (0.325–0.326 nm). Conversely, for [*t*-BOC(oxy), *t*-BOC(oxy)], the *anti*-structure is well packed with substituents, showing a shorter distance between benzene rings (0.327 nm) compared to that for the *syn*-structure (0.338 nm). Despite the predicted decrease in π-π interactions with increasing distance between phenyl rings in the *syn*-structure, the Gibbs free energy for the *syn*-structure (Δ*G*^0^ =  − 0.008 eV) is similar to that for the *anti*-structure. The pEDA calculations for the dimer cation model of (OH, OH) reveal a small electron-donating behavior, with values of 0.024 and 0.03 for both conformations. By contrast, [*t*-BOC(oxy), *t*-BOC(oxy)] displayed an electron-accepting behavior with pEDA of − 1.284 (*anti*) and − 0.256 (*syn*). Similar results were observed for the neutral model, where (OH, OH) displayed an electron-donating behavior and [*t*-BOC(oxy), *t*-BOC(oxy)] displayed electron-accepting behavior. The lower ionization potential of the (OH, OH) model compared to that of the styrene dimer model can be explained by the electron-donating behavior of OH groups. However, [*t*-BOC(oxy), *t*-BOC(oxy)] has a lower ionization potential despite its electron-accepting behavior. This cannot be explained solely by substituent effects on π– π interactions, suggesting that bulky substituent interactions contribute to the stabilization of the cation formation. Contrary to the monomer cation model, the conversion of the substituent effect to electron-accepting behavior may be due to the increased electron density to the substituent with the increased interaction between the *t*-BOC(oxy) groups, as shown in Figs. S5 and S6. The total NBO charges of the *t*-BOC(oxy) groups in the dimer cation model obtained are [− 0.129 (*anti*)] and [− 0.100 (*syn*)], which are smaller than the value for the monomer cation (0.066).

Next, a model of a heterodimer radical cation substituted with *t*-BOC(oxy) and hydroxyl groups [*t*-BOC(oxy), OH] is discussed. The converged structure is shown in Fig. 6C**.** This structure exhibits a clear hydrogen bond between the H atom of the OH group and the carbonyl oxygen of the *t*-BOC group, which may contribute to the stabilization of the cation. The [*t*-BOC(oxy), OH] model has a negative pEDA value of − 0.148, indicating an electron-accepting behavior, similar to the [*t*-BOC(oxy), *t*-BOC(oxy)] group. The positive charge in the heterodimer radical cation is mainly localized to the vinylphenol unit, as indicated by the NBO charge and electron density maps. This suggests that deprotonation from the vinylphenol unit is likely to occur after charge equilibrium in the copolymer radical cation. Due to the stabilization of the cation by hydrogen bonding, the ionization energy of the heterodimer model is lower than that of the PS model and also the lowest compared to the PTBOS and PHS dimer models (Table 1). This implies that the localization of holes to the heterodimer moiety in the copolymer is more likely than to the homodimer moiety. In contrast, the probability of the presence of heterodimers in the diad in polymers reaches a maximum at 50% protection (Figure S8), corresponding to an extremum in the acid yield and a deprotonation rate constant around 50% protection. The presence of intramolecular hydrogen bonds as well as the reduction of π–π interactions, may contribute to the instability of the cation, causing an effect that promotes the deprotonation reaction. Therefore, it is suggested that the enhancement of the deprotonation via intramolecular hydrogen bonding is a factor contributing to the high acid yield and decay rate constant at 50% protection (Figs. 4, 5). As mentioned earlier, the acid yield in PTBOS film is approximately 80% of that of the PHS film, which is relatively high despite the absence of proton-releasing OH groups^40^. The low deprotonation efficiency in the PS film^41^ suggests that the main deprotonation source is the *t*-BOC(oxy) substituent. Thus, the high deprotonation efficiency of PTBOS may primarily be caused by intra- and intermolecular hydrogen bonding based on interactions between substituents. In addition, interactions between the bulky *t*-BOC groups may act as an electron-accepting substituents, thereby reducing π-electronic interactions and triggering deprotonations due to the reduced stability of the radical cation.

Finally, the CR band transitions calculated by TD-DFT indicated that the transitions for PHS and PTBOS dimer cations are similarly predicted to be around 1100 nm. This observation is consistent with the pulse radiolysis results. Furthermore, the oscillator strength is greater for the (OH, OH) dimer model than for the [*t*-BOC(oxy), *t*-BOC(oxy)] model, which is consistent with the pulse radiolysis result that the absorption intensity of the CR band increases with the percentage of the vinylphenol unit as shown in Fig. 2c and d**.** However, [*t*-BOC(oxy), OH] does not show a clear CR band in the NIR region, owing to hole localization in the vinylphenol unit. Pulse radiolysis showed the lowest CR band absorption is for the *n* = 0.55 polymer as shown in Fig. 2b, suggesting a significant contribution of heterodimer radical cations in the copolymerization ratio polymer close to 50%.

## Conclusions

Pulse radiolysis of poly(4-hydroxystyrene) (PHS)–type polymer solutions was performed to investigate how the dynamics of the radical cation depend on the copolymerization ratio. It was demonstrated that the deprotonation rate of *tert*-butoxycarbonyl (*t*-BOC)–protected polymers tends to decrease with increasing protection ratio. However, the copolymer with a copolymerization ratio of 50% specifically shows a faster deprotonation reaction and a higher acid yield. This phenomenon can be attributed to intramolecular hydrogen bonding in heterodimer cations, as shown by the quantum chemical calculations of the model molecules. In addition, poly(4-(*tert*-butoxycarbonyl)oxy)styrene shows a relatively high acid yield of 80% compared to PHS after ionization. While the *t*-BOC(oxy) group serves as an electron donor in the monomer radical cation, it switches to electron-accepting in the radical cation formed in the polymer, owing to the increased electron density on the substituents. The presence of hydrogen bonds between intra- and intermolecular *t*-BOC groups, as well as the reduction of π-π interactions, may contribute to the instability of the cation, causing an effect that promotes the deprotonation reaction. As a design guideline for base polymers of extreme ultraviolet and electron-beam chemically amplified resists, structures that promote intramolecular hydrogen bonding and reduce *π*-*π* interactions of cations are considered to be effective in improving the quantum yield of acids.

## Data Availability

The authors confirm that the data supporting the findings of this study are available within the article and its supplementary materials.

## Supplementary Figures

Supplementary Figures.
