# Supplementary material for: Dynamics of ionized poly(4-hydroxystyrene)-type resist polymers with tert-butoxycarbonyl-protecting group
Source: Sci Rep. 2024 Jul 20;14:16729. doi: 10.1038/s41598-024-67794-0 (PMC11271537; doi:10.1038/s41598-024-67794-0)
Supplement: Supplementary file 1 — Supplementary Figures. [file 41598_2024_67794_MOESM1_ESM.docx]

**Supplementary Information**

Kazumasa Okamoto,^1,2*^ Yusa Muroya,^1^ Takahiro Kozawa^1^

**Dynamics of ionized poly(4-hydroxystyrene)-type resist polymers with *tert*-butoxycarbonyl-protecting groups**

1. SANKEN (The Institute of Scientific and Industrial Research), Osaka University, Ibaraki, Osaka 567-0047, Japan

2. Artificial Intelligence Research Center, SANKEN (AIRC-SANKEN), Osaka University, Ibaraki, Osaka 567-0047, Japan

E-mail: kazu@sanken.osaka-u.ac.jp

**Fig. S1.** Kinetic traces obtained by nanosecond pulse radiolysis at 600 nm: (a) PTBOS (100 mM (unit conc.)) in Ar-saturated CHN, (b) PTBOS (100 mM (unit conc.)) with 5 mM TPS-nf in Ar-saturated CHN.

**Fig. S2.** FT-IR spectra of OH vibrations of PHS and P(TBOS-*co*-HS) films on an undoped Si wafer with *t*-BOC protection ratios *n* of 0.55 and 0.76.


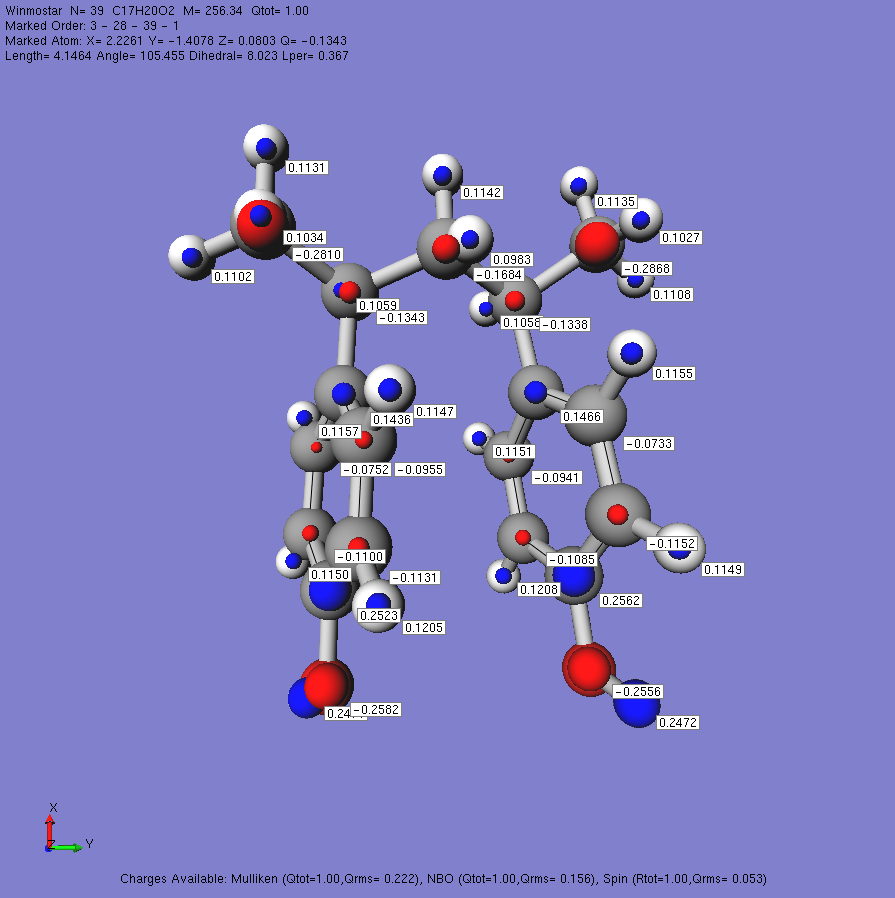


PHS (anti) NBO


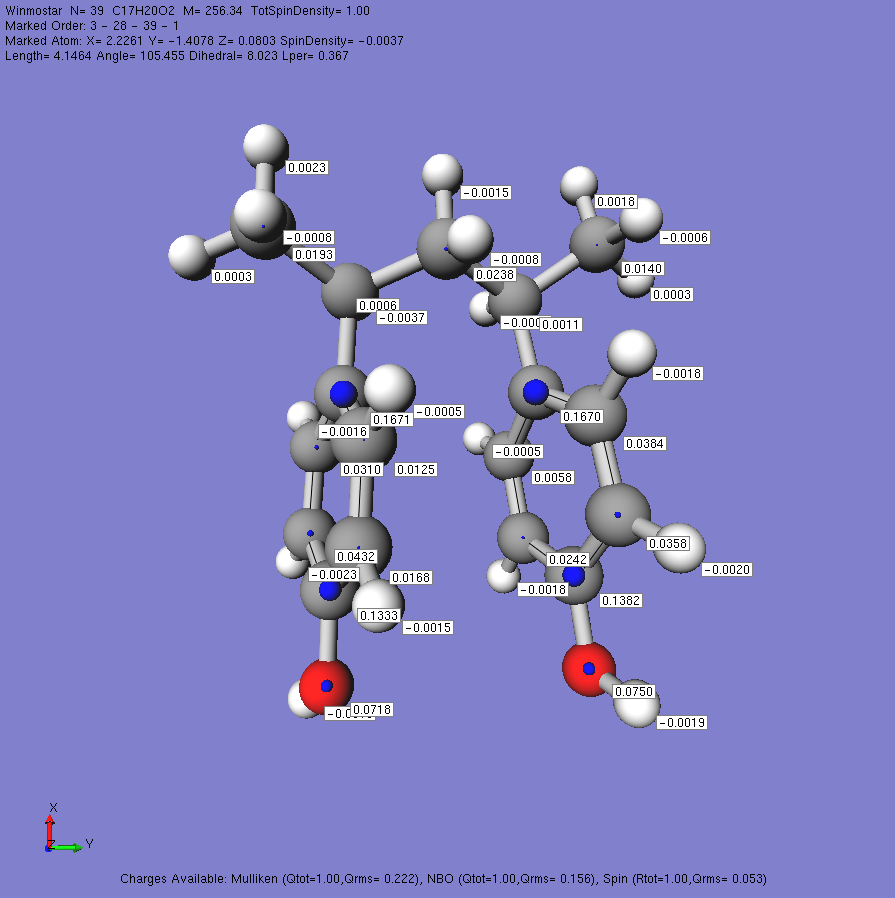


PHS (anti) spin

**Fig. S3.** Optimized structure of *m*-2,4-diphenyl pentane derivatives as the PHS dimer radical cation model (OH, OH) with NBO charge and spin density on atoms. The hydroxyl groups are in the opposite direction (*anti*). DFT calculations are carried out using the wB97X-D/def2-TZVP level.


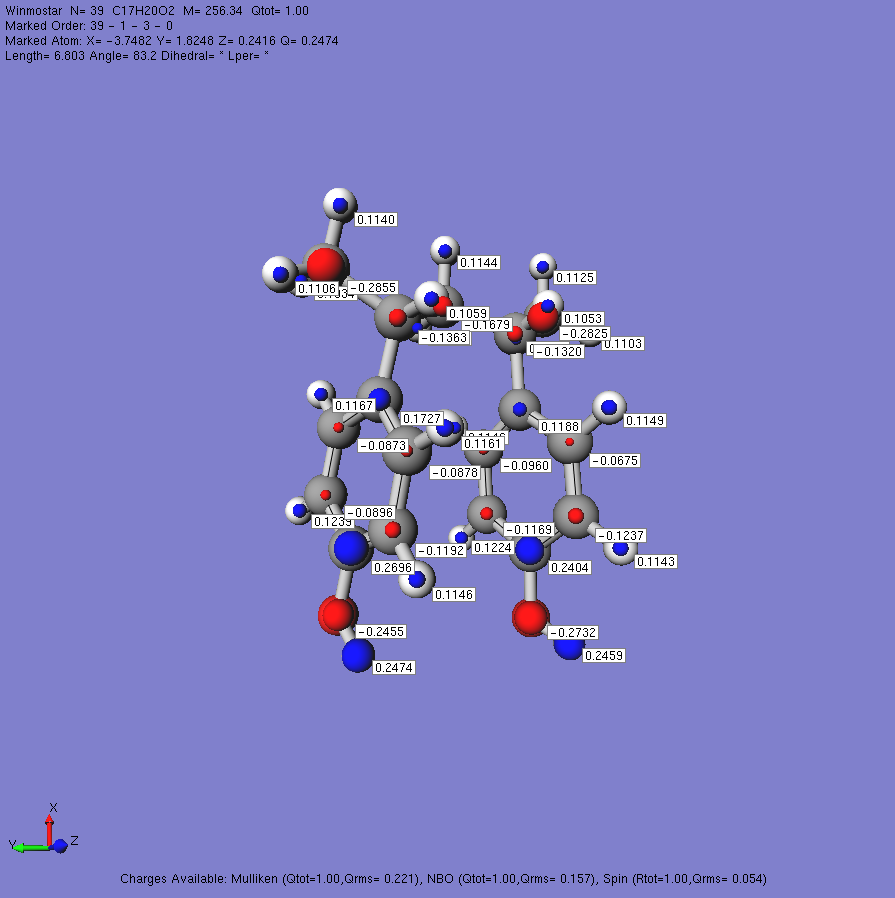


PHS (syn) NBO


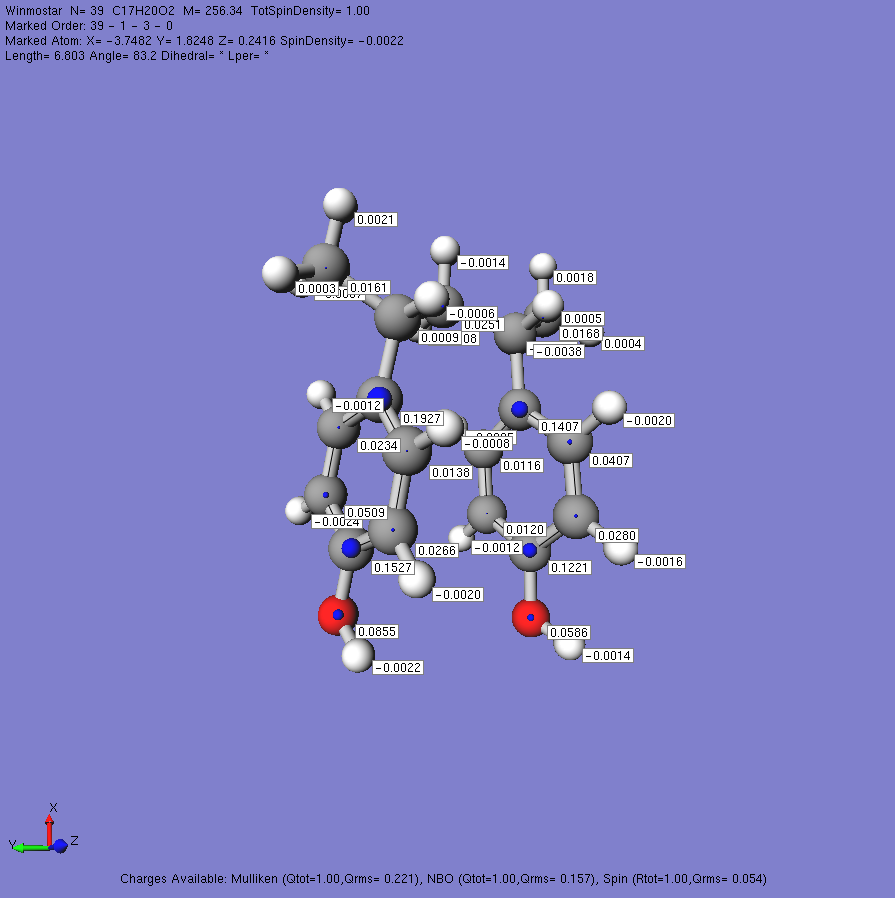


PHS (syn) spin

**Fig. S4.** Optimized structure of *m*-2,4-diphenyl pentane derivatives as the PHS dimer radical cation model (OH, OH) with NBO charge and spin density on atoms. The hydroxyl groups are in the same direction (*syn*). DFT calculations are carried out using the wB97X-D/def2-TZVP level.


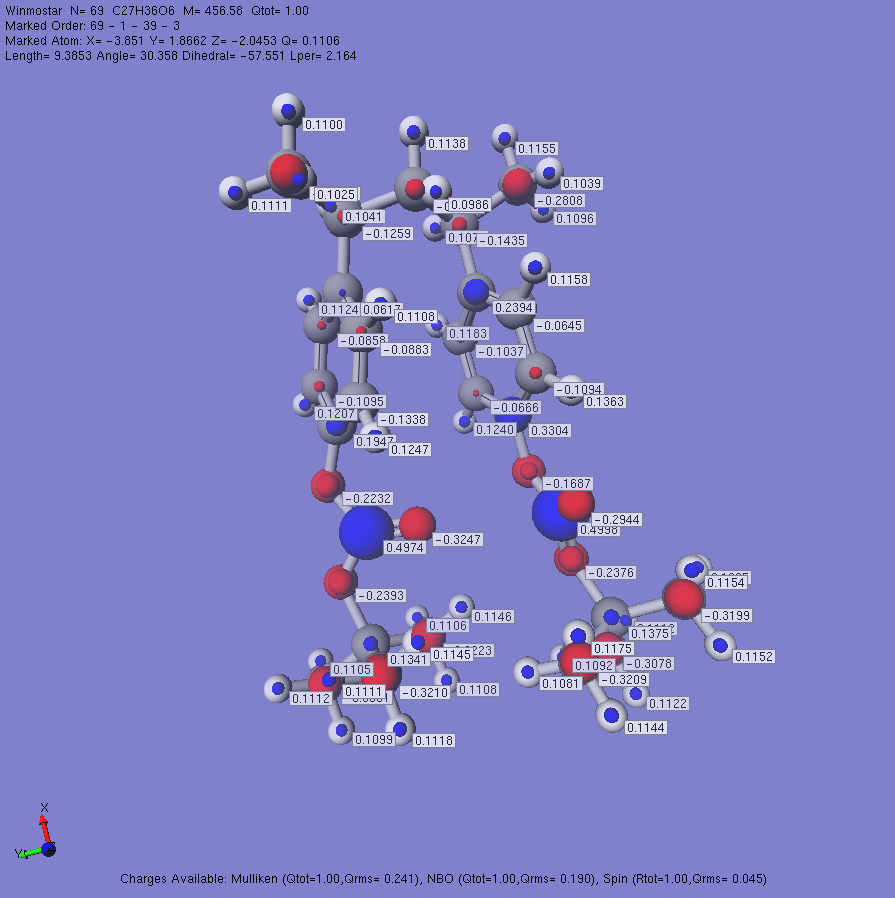


PTBOS (*syn*) NBO


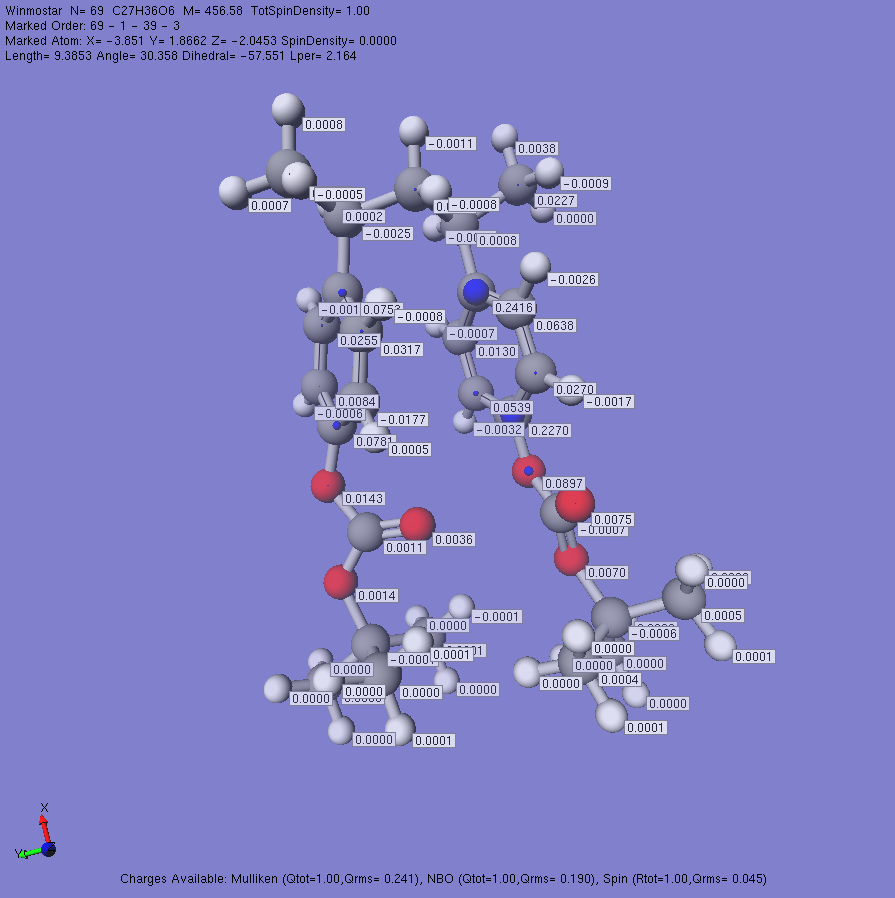


PTBOS (*syn*) spin

**Fig. S5.** Optimized structure of *m*-2,4-diphenyl pentane derivatives as the PTBOS dimer radical cation model (*t*-BOC, *t*-BOC) with NBO charge and spin density on atoms. The carbonyl O atom of *t*-BOC groups are in the same direction (*syn*). DFT calculations are carried out using the wB97X-D/def2-TZVP level.


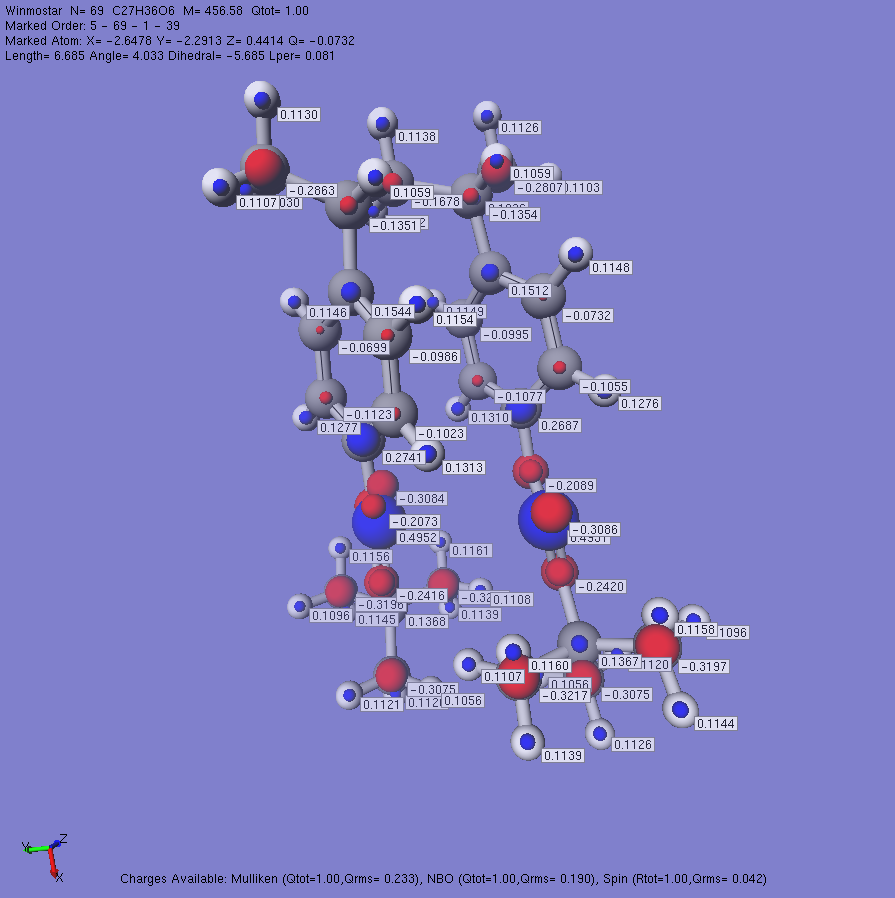


PTBOS (*anti*) NBO


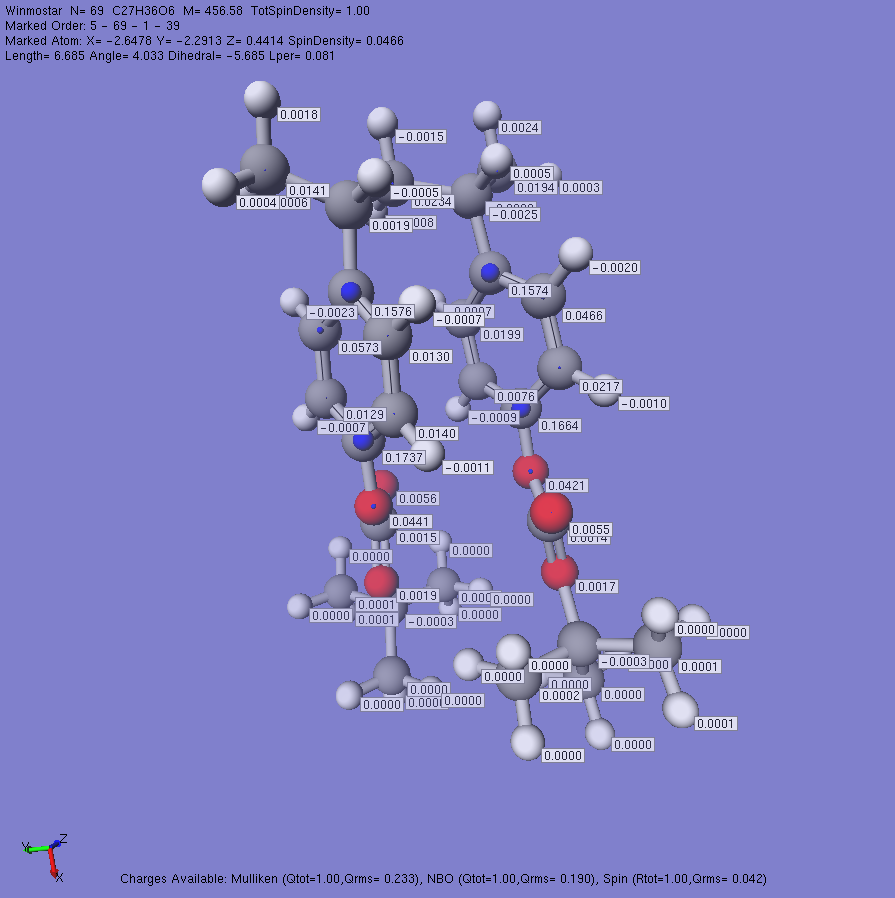


PTBOS (*anti*) spin

**Fig. S6.** Optimized structure of *m*-2,4-diphenyl pentane derivatives as the PTBOS dimer radical cation model (*t*-BOC, *t*-BOC) with NBO charge and spin density on atoms. The carbonyl O atom of *t*-BOC groups are in the same direction (*anti*). DFT calculations are carried out using the wB97X-D/def2-TZVP level.


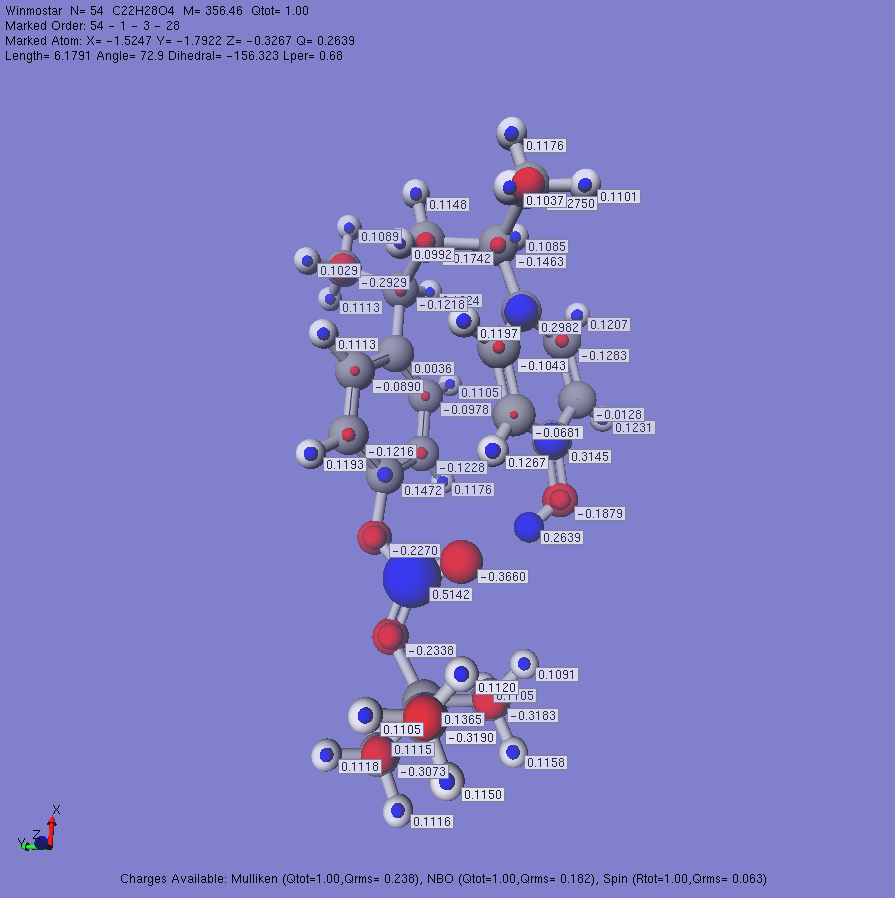


P(TBOS-*co*-HS) NBO


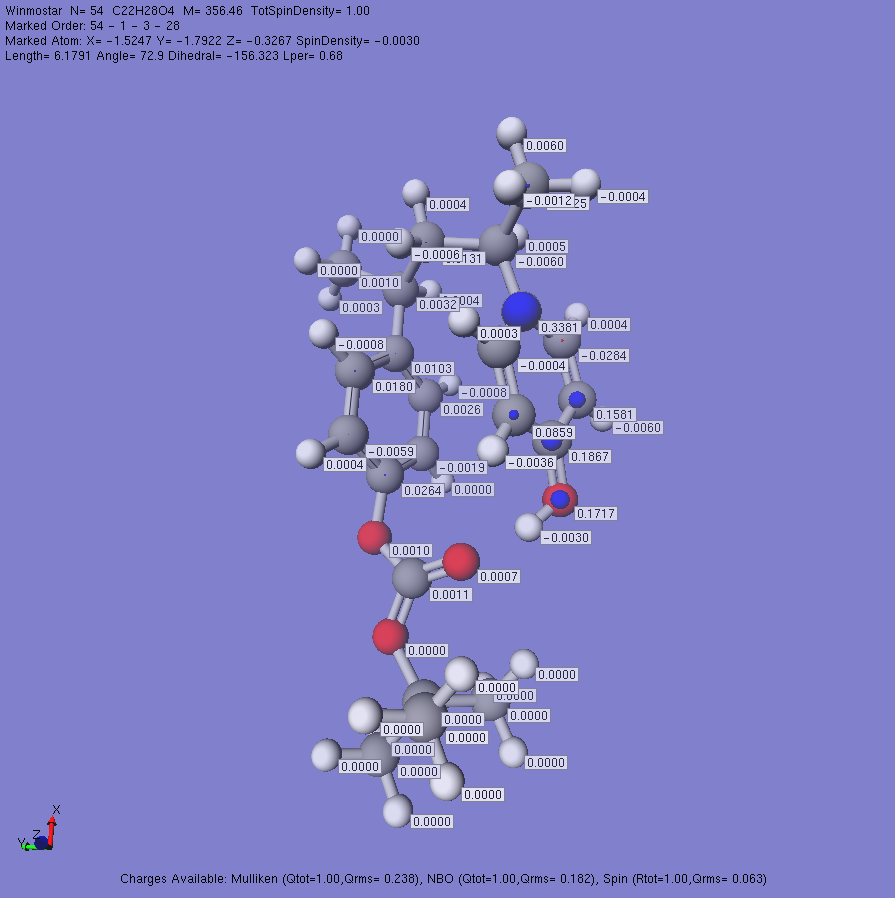


P(TBOS-*co*-HS) spin

**Fig. S7.** Optimized structure of *m*-2,4-diphenyl pentane derivatives as the P(TBOC-*co*-HS) dimer radical cation model (*t*-BOC, OH) with NBO charge and spin density on atoms. DFT calculations are carried out using the wB97X-D/def2-TZVP level.

**Fig. S8.** Relationship between the probability of presence of hydroxystyrene units and *t*-BOC-protected hydroxystyrene units in the diad and the protection ratio based on the Bernoulli model [1]. Here, the combinations of substituents on the benzene ring are HH: (OH, OH), HT: (*t*-BOC(oxy), OH), and TT: (*t*-BOC(oxy), *t*-BOC(oxy)).

**Reference**

[1] Smith, L. M. & Coote, M. L. Effect of temperature and solvent on polymer tacticity in the free-radical polymerization of styrene and methyl methacrylate. *Polym. J.* **51,** 3351–3358 (2013).
